# Supplementary material for: Bottlenecks, Modularity, and the Neural Control of Behavior
Source: Front Behav Neurosci. 2022 Apr 6;16:835753. doi: 10.3389/fnbeh.2022.835753 (PMC9020368; doi:10.3389/fnbeh.2022.835753)
Supplement: Supplementary file 1 [file Data_Sheet_1.PDF]

Supplementary Material

1 SUPPLEMENTARY TABLES AND FIGURES

1.1 Figures

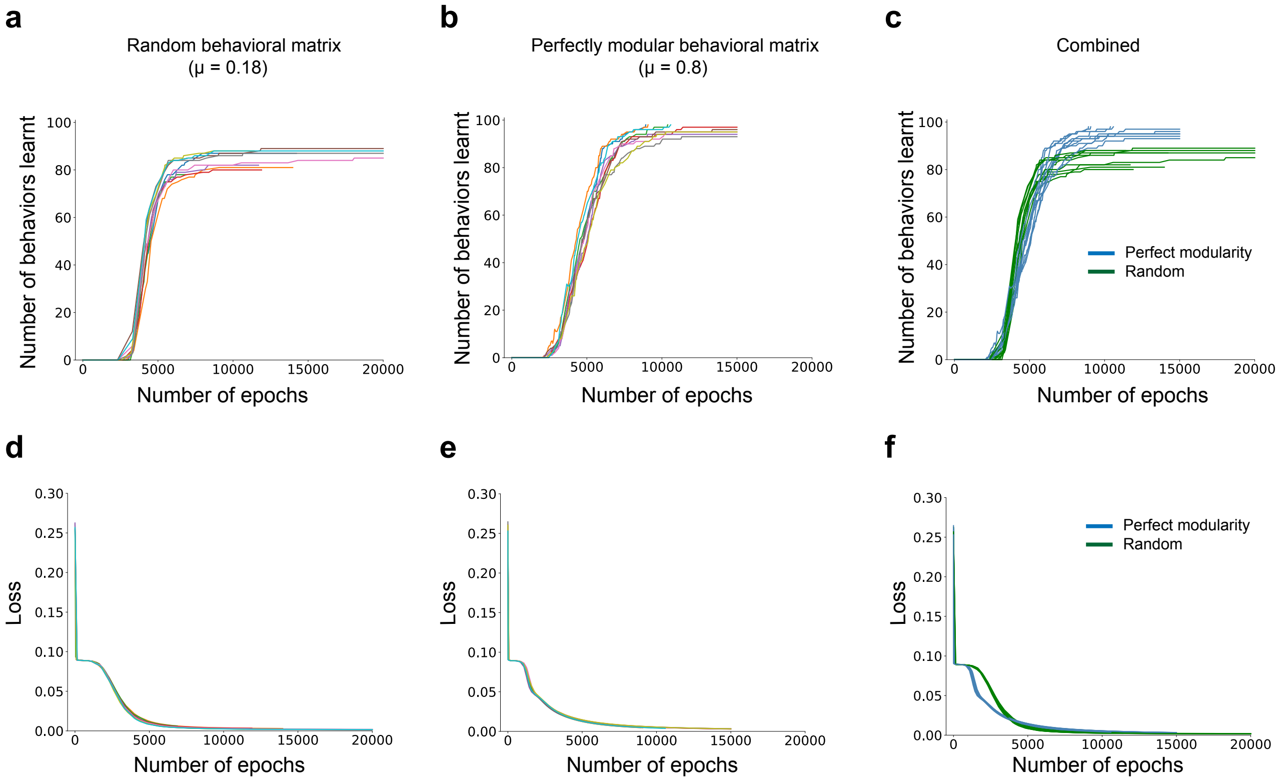

Figure S1. Learning and loss curves. Learning **(a)-(c)** and loss curves **(d)-(f)** obtained while training the network when the behavioral matrix is **(a)&(d)** random ($\mu = 0.18$), **(b)&(e)** perfectly modular ($\mu = 0.8$) with the combined results in **(c)&(f)**. $N = M = 100$ with $R = 25$ for the random and $R = 13$ for the fully modular case. For each value of modularity we plot n=10 curves, with the different colors corresponding to the different iterations.

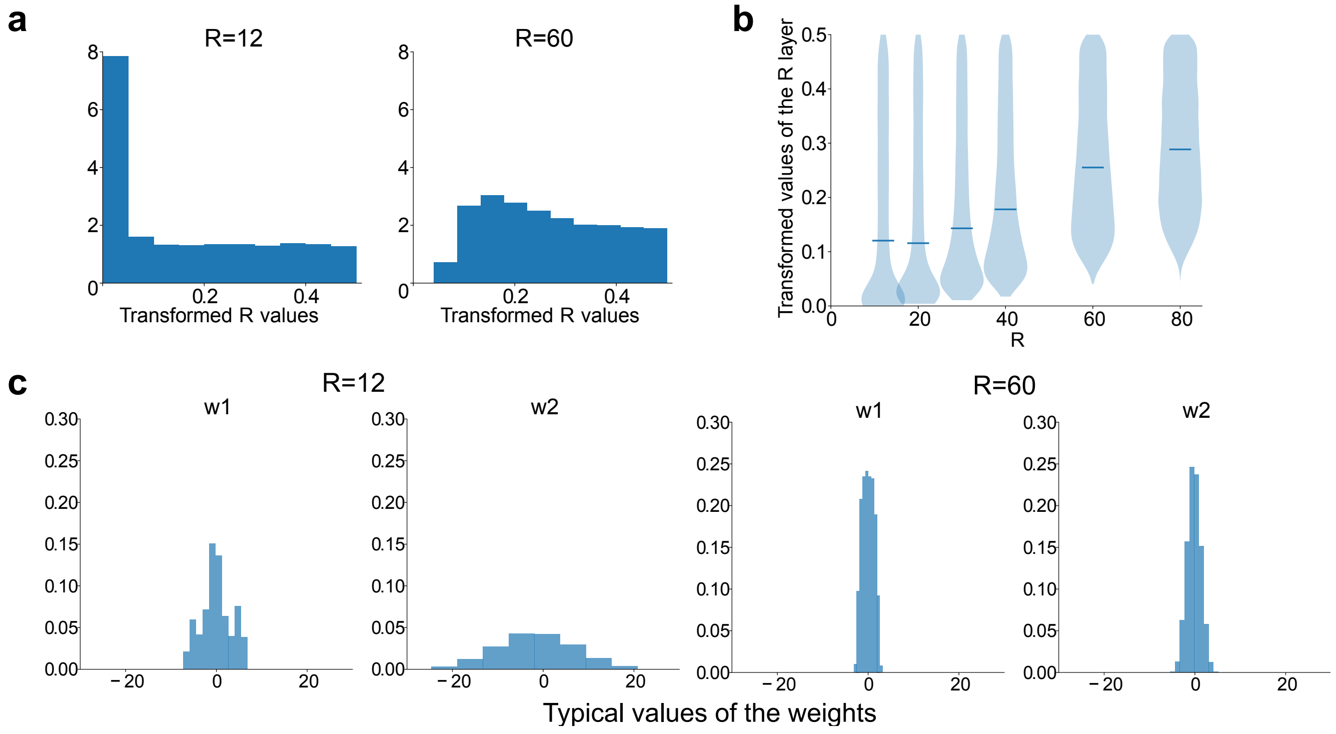

Figure S2. Typical values of the hidden layer and the weights of the trained network. (a) Histogram of typical values of the hidden layer for $R = 12$ (smaller than the critical bottleneck size) and $R = 60$ (larger than the critical bottleneck size). Values lie between 0 and 1 but here we map the values in the $1 - 0.5$ interval to the $0 - 0.5$ interval to highlight the extent to which the hidden layer is binarized, with more values close to 0 corresponding to more binarization. **(b)** Violin plots of the transformed values of the hidden layer as a function of the size of the hidden layer. Horizontal bars indicate median values. **(c)** Typical weights of the trained network for $R = 12$ and $R = 60$. These results correspond to network parameters $N = M = 100$ and $k = 10$

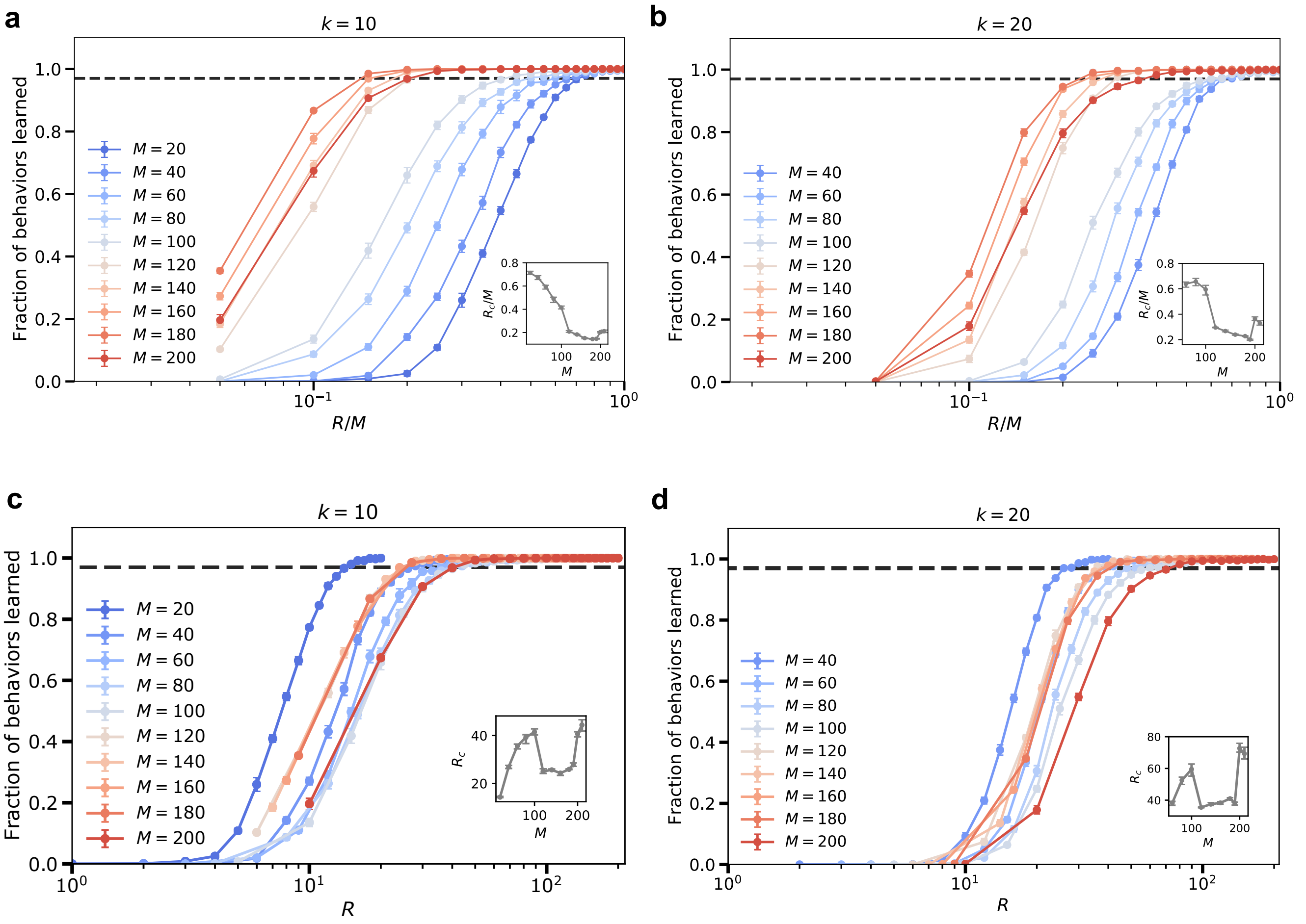

Figure S3. Network characterization. (a)-(b) Fraction of behaviors learned as a function of hidden layer size R and fixed input layer size $N = 100$ for varying output layer size M and fixed number of active output neurons ($k = 10$ (a) and $k = 20$ (b)). The horizontal axis is the hidden layer size scaled by the output layer size R/M. Insets show the scaled critical bottleneck size R_c/M as a function of M. Each point is averaged over 30 random input-output combinations. Dashed line indicates critical bottleneck threshold. **(c-d)** Subfigures (a-b) with the horizontal axis replaced by the unscaled hidden layer size R. The curves loosely collapse onto each other; this allows us to use one value of M for most of this work. The insets show the critical bottleneck size R_c as a function of M. For $k = 10$, the network with output layer size of $M = 100$ has the most difficulty learning (highest threshold bottleneck size R_c). We thus focus our studies on $M = 100$ networks for $k = 10$ sparsity signals in the main text.

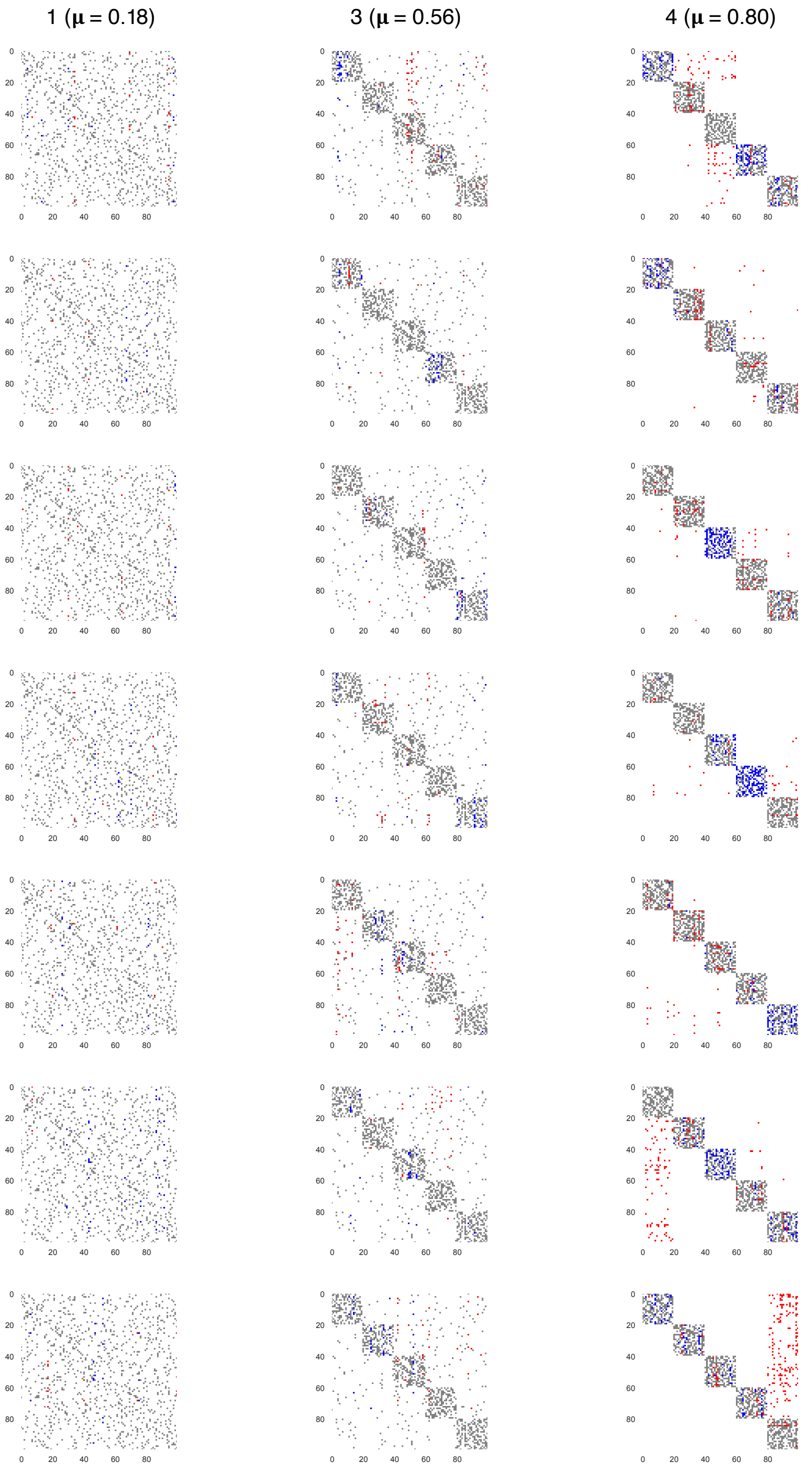

Figure S4. Hidden layer perturbations. Other examples of a hidden layer perturbation on the networks trained for behavior matrices with different modularities at R_c point. Numbers correspond to the point numbers in panel a. In each case, one of the hidden neurons is kept constantly activated, while the rest of the network tried to reproduce the original set of behaviors. White and grey colors correspond to unperturbed motor neurons, non-active and active correspondingly. Blue indicates motor units that have been deactivated for different behaviors due to this hidden neuron activation. Conversely, red shows motor units that have been activated.

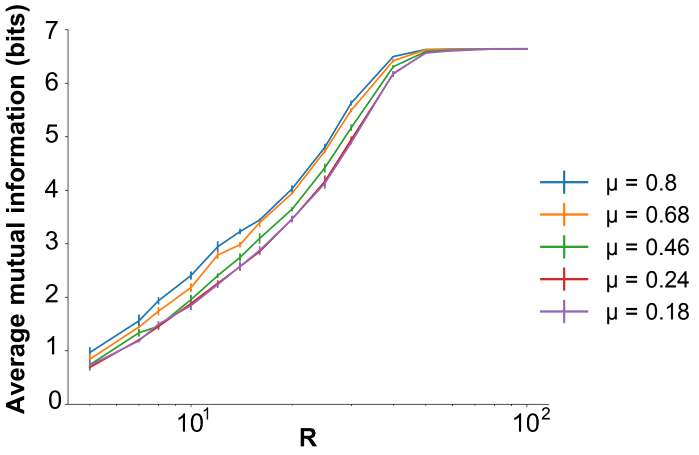

Figure S5. Mutual information as a function of the hidden layer size Average mutual information between the input and output distributions after forced activation of each hidden neuron as a function of the size of the hidden layer R, and varying levels of modularity, μ. $N = M = 100$ and results are means over 5 iterations with the error bars corresponding to the standard deviation.

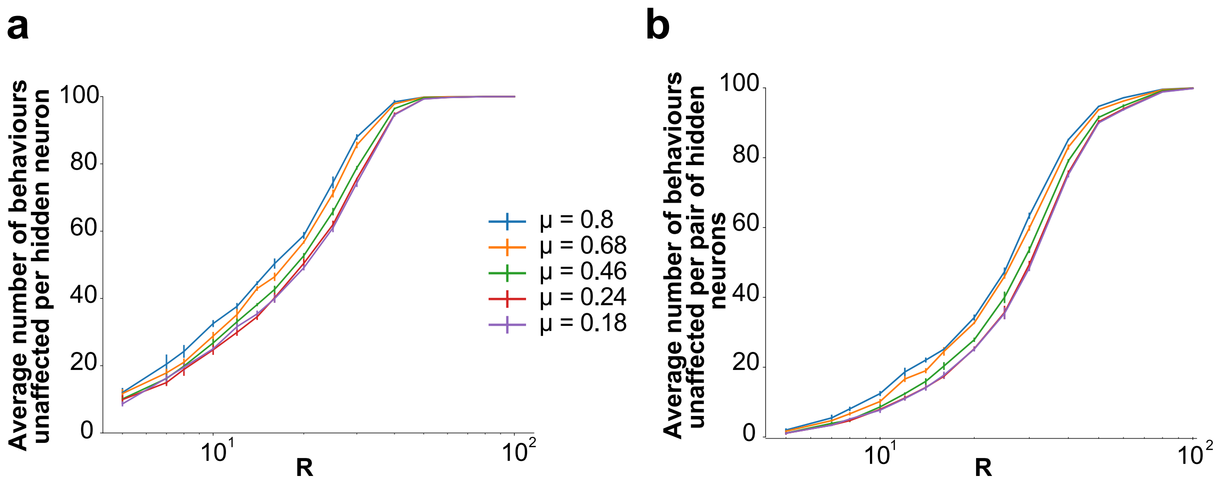

Figure S6. Robustness upon deactivation and double activation. **(a)** Robustness of the network averaged over the effects of de-activating each hidden neuron as a function of the hidden layer size, R and varying levels of modularity, μ. **(b)** Robustness of the network averaged over the effects of activating a pair of hidden neurons as a function of the hidden layer size, R and varying levels of modularity, μ. **(a)-(b)** $N = M = 100$ and results are means over 5 iterations with the error bars corresponding to the standard deviation.

1.2 Tables

μ (mean ± std)	R_c (mean ± std)
0.180 ± 0.004	41.6 ± 1.7
0.369 ± 0.003	41.0 ± 2.1
0.464 ± 0.003	38.2 ± 1.9
0.566 ± 0.002	33.8 ± 1.4
0.677 ± 0.002	25.9 ± 1.4
0.800 ± 0.0001	13.4 ± 0.9

Table S1. Critical bottleneck size. Values of the critical bottleneck size R_c for different values of modularity μ. The mean and standard deviation are calculated over 10 experiments with different output matrices for each value of modularity.
